# IgG4/IgG RNA ratio does not accurately discriminate IgG4-related disease from pancreatobiliary cancer

**DOI:** 10.1016/j.jhepr.2020.100116

**Published:** 2020-04-14

**Authors:** Elsemieke de Vries, Floor Tielbeke, Lowiek Hubers, Jeltje Helder, Nahid Mostafavi, Joanne Verheij, Jeanin van Hooft, Marc Besselink, Paul Fockens, Niek de Vries, Ulrich Beuers

**Affiliations:** 1 Department of Gastroenterology & Hepatology, The Amsterdam Gastroenterology & Metabolism (AG&M) Research Institute, The Netherlands; 2 Department of Pathology, The Amsterdam Gastroenterology & Metabolism (AG&M) Research Institute, The Netherlands; 3 Department of Surgery, Cancer Center Amsterdam, The Netherlands; 4 Department of Rheumatology & Clinical Immunology, all at Amsterdam UMC, University of Amsterdam, The Netherlands

**Keywords:** Cholangiocarcinoma, IgG4-related cholangitis, Klatskin tumor, pancreatic carcinoma, pancreatobiliary malignancy, AIP, auto-immune pancreatitis, AUC, area under the curve, CA 19.9, carbohydrate antigen 19.9, HPF, high power field, IgG4-RD, IgG4-related disease, IRC, IgG4-related cholangitis, PSC, primary sclerosing cholangitis, ROC, receiver operating characteristics

## Abstract

**Background & Aims:**

IgG4-related disease (IgG4-RD) of the biliary tract and pancreas is often difficult to distinguish from pancreatobiliary cancer. The blood IgG4/IgG RNA ratio has been reported to discriminate IgG4-RD from primary sclerosing cholangitis/pancreatobiliary cancer with high accuracy. This study aimed to prospectively assess the diagnostic accuracy of the blood IgG4/IgG RNA ratio for distinguishing IgG4-RD from cancer in patients with a suspected pancreatobiliary malignancy.

**Methods:**

In this prospective, single center, observational study, patients presenting at a specialized multidisciplinary, hepato-pancreato-biliary clinic with suspicion of pancreatobiliary malignancy were included. The IgG4/IgG RNA ratio (threshold 5.0%) was determined by quantitative PCR in addition to standard diagnostic procedures. Clinical, biochemical, radiological, and histo-/cytopathological findings were analyzed. For the diagnosis of IgG4-RD, the HISORt criteria were used as a reference standard. Malignancy was defined by the presence of neoplastic tissue at histo-/cytopathological examination.

**Results:**

Overall, 213 consecutive patients (mean age 68 years) with a suspected pancreatobiliary malignancy were analyzed, of whom 3 patients were diagnosed with IgG4-RD and 178 patients were diagnosed with malignancy (165 patients with primary pancreatobiliary malignancy). The IgG4/IgG RNA ratio was true positive in 3 patients and false positive in 87 (40.8%) patients. In 123 (57.7%) patients the test was true negative. The sensitivity of blood IgG4/IgG RNA ratio was 100%, the specificity 58.6%, the positive predictive value 3.3%.

**Conclusion:**

In the setting of a high *a priori* risk of malignancy, an elevated IgG4/IgG RNA ratio did not accurately discriminate pancreatobiliary cancer from IgG4-RD as illustrated by low specificity and concordant low positive predictive value. We advise against the use of this test to discriminate IgG4-RD from pancreatobiliary malignancies.

**Lay summary:**

IgG4-related disease is a benign inflammatory multiorgan disease which predominantly affects the pancreas and biliary tree. Clinical symptoms, laboratory and imaging finding are often difficult to distinguish from pancreatic or biliary tract cancer. This prospective trial indicates that the recently proposed blood IgG4/IgG RNA ratio does not accurately distinguish benign IgG4-RD from malignant pancreatobiliary disease.

## Highlights

• IgG4-related disease and malignancy of bile ducts/pancreas can be indistinguishable.
• Blood IgG4/IgG RNA ratio was prospectively tested as an IgG4-RD marker when malignancy was suspected.
• IgG4/IgG RNA ratio >5% did not accurately discriminate IgG4-RD from pancreatobiliary cancer.

## Introduction

Immunoglobin G4-related disease (IgG4-RD) is a systemic immune-mediated disease which affects various organs, predominantly the pancreas, bile ducts and salivary glands.^1^ IgG4-RD of the pancreas (auto-immune pancreatitis [AIP] type 1) and the bile ducts (IgG4-related cholangitis, IRC) are benign inflammatory diseases which are responsive to corticosteroid treatment.^2^^,^^3^ Diagnostic criteria such as the HISORt criteria including histopathological (H), imaging (I), serological (S) features, other organ manifestations of IgG4-RD (O) and response to treatment (Rt), have been developed to diagnose pancreatobiliary IgG4-RD.^3^ Still, the accuracy of these diagnostic criteria is limited and AIP type I and IRC remain difficult to distinguish from pancreatobiliary malignancies or primary/secondary sclerosing cholangitis.^4^^,^^5^ Clinical signs and symptoms of obstructive jaundice, weight loss and abdominal discomfort are frequently seen in AIP and IRC as well as in pancreatobiliary malignancies. On imaging, pancreatic mass and biliary strictures or mass-forming lesions are seen in both conditions as well.^3^ Unfortunately, misdiagnosis is common and may result in unnecessary surgical interventions or systemic chemotherapy in patients with IgG4-RD. The rate of unexpected benign disease after hemihepatectomy for presumed cholangiocarcinoma or pancreatoduodenectomy for presumed pancreatic ductal carcinoma still ranges from 5 to 15%,[6], [7], [8] with IgG4-RD being a major contributor.^8^ Remarkably, up to a third of patients with IgG4-RD may undergo major surgery due to presumed malignancy prior to an accurate diagnosis of IgG4-RD.^9^ IgG4 serum levels have limited diagnostic value when only modestly elevated (<4x the upper limit of normal), since 10–15% of patients with pancreatobiliary cancers and primary sclerosing cholangitis (PSC) may also exhibit increased IgG4 serum levels.[10], [11], [12] Moreover, up to 30% of patients with IgG4-related pancreatobiliary disease have normal serum IgG4 levels.^3^^,^^13^ Histo- or cytopathological confirmation of malignancy is often difficult to obtain, particularly of a hilar mass, and histological differentiation between benign and malignant pancreatobiliary entities can be challenging. Elevated carbohydrate antigen (CA) 19.9 level is not sufficient to differentiate between benign and malignant pancreatobiliary disease with obstructive jaundice.^14^

Our group recently proposed blood IgG4/IgG RNA ratio, determined by quantitative PCR (qPCR), as a diagnostic test to discriminate IgG4-RD from PSC or pancreatobiliary cancer^12^ based on the high accuracy (94% sensitivity; 99% specificity) of this test for distinguishing patients with IgG4-RD from those with PSC and biliary/pancreatic cancer.^12^ The present study aimed to detect IgG4-RD in patients presented consecutively with suspected biliary or pancreatic cancer, in order to prevent misdiagnosis by determining the blood IgG4/IgG RNA ratio by qPCR for the diagnosis of IgG4-RD.

## Patients and methods

In this prospective, observational, single center study, all consecutive patients of ≥18 years of age presenting at a specialized multidisciplinary hepato-pancreato-biliary clinic, at the Amsterdam UMC, with suspected or proven biliary or pancreatic cancer were included between February 21^st^ and September 4^th^, 2019. Standard diagnostic procedures were performed including laboratory assessment (hemoglobin, mean corpuscular volume, leukocytes, erythrocytes, platelets, creatinine, albumin, blood urea nitrogen, glucose, potassium, sodium, total bilirubin, alkaline phosphatase, gamma-glutamyltransferase, alanine aminotransferase, aspartate aminotransferase, prothrombin time, CA19.9, IgG and IgG4), radiographic imaging (ultrasound, CT and/or MRI) and histo-/cytopathological analysis. In addition, the blood IgG4/IgG RNA ratio was determined and considered positive if the ratio was >5.0%.^12^

The exact method for determining the IgG4/IgG RNA ratio has been extensively described.^12^ In brief, RNA was isolated from peripheral blood and processed. Complementary DNA (cDNA) was synthesized from RNA using Superscript III RT (Invitrogen Life Technologies, Carlsbad, CA). IgG and IgG4 primers for qPCR were designed to specifically amplify sequences that encode the constant region of the heavy chain of the receptor. Additionally, the following rules were applied: (1) 2 different reverse primers were designed, one for all possible IgG subtypes and one specific for the IgG4 subtype, and (2) the 2 reverse primers were designed on virtually the same position to allow comparable conditions. After optimization, this resulted in 1 universal forward primer (5′-GCTGCCTGGTCAAGGACTAC-3′), 1 generic IgG reverse primer (5′-TCTTGTCCACCTTGGTGTTG-3′), and 1 specific IgG4 reverse primer (5′-CTACGTTGCAGGTGTAGGTCTTC-3′). Duplicate qPCR reactions were performed in a 10 μl total volume in the presence of 10 pmol of the forward and reverse primers, cDNA from 50 ng RNA input, and 5 μl of SensiFAST SYBR Lo-ROX reagent (catalog no. BIO-94005, Bioline; GC Biotech, Alphen aan den Rijn, Netherlands) for 40 cycles (95°C for 2 minutes, 40 cycles [95°C for 5 seconds, 60°C for 10 seconds, and 72°C for 20 seconds], followed by a melting curve [95°C for 5 seconds, 65°C for 1 minute, and 97°C continuous]), using the LightCycler 480 system (Roche Diagnostics, Almere, The Netherlands). Both for IgG and IgG4, the starting concentrations in the sample were calculated using LinRegPCR software15 and used to calculate the percentage of IgG+ RNA molecules that were IgG4+.^12^

Clinical, laboratory, imaging and histo-/cytopathological findings were prospectively collected from all patients investigated and treated. Patients were discussed in a weekly multidisciplinary meeting in the presence of at least 1 surgeon, gastroenterologist, oncologist, pathologist and radiologist. Histo-/cytopathological findings were analyzed in patients who underwent a biopsy, brush or surgical intervention of the biliary tract or pancreas. For the diagnosis of IgG4-RD, the HISORt criteria were used as a reference standard.^15^ Malignancy was defined by the presence of neoplastic tissue/cells at histo-/cytopathological examination. Patients with high suspicion of IgG4-RD and no proven malignancy, were treated with prednisolone (30–40 mg daily) for 2–4 weeks and were then re-evaluated by laboratory tests and imaging to monitor tumor response.

### Sample size calculation

Every year approximately 450–500 patients are seen at the specialized outpatient hepato-pancreato-biliary clinic. Based on the assumption that every year circa 5 new patients with IgG4-RD will present, 1,000 patients had to be screened in 2 years to identify 10 patients with IgG4-RD. In order to evaluate the predictive value of the clinical decision model to distinguish between IgG4-RD of the biliary tract or pancreas and cancer, a minimum of 10 patients with IgG4-RD had to be tested, to allow for 1 false negative result, based on a sensitivity of 94% and a specificity of 99% of the IgG4/IgG RNA ratio. Using the IgG4/IgG RNA ratio alone, we estimated to have 1 false negative and 10 false positive results. However, when combining the accuracy of blood IgG4/IgG RNA ratio for IgG4-RD with brush cytology for cholangiocarcinoma and pancreatic carcinoma (sensitivity 30–50%, specificity 99%), EUS-FNA for pancreatic carcinoma (sensitivity 80%, specificity 100%) and biopsies of presumed metastases, we aimed to lower the number of false positive test results from 10 to 3. On the other hand, 9 patients would be diagnosed correctly with IgG4-RD and would be saved from unnecessary surgery or chemotherapy.

An interim analysis of this open, prospective, single center trial was performed in September 2019 and the study was subsequently stopped on September 10, 2019.

### Statistical analysis

The predictive value of the blood IgG4/IgG RNA ratio was calculated with a true positive test defined as an elevated IgG4/IgG RNA ratio ≥5% in combination with response to prednisolone treatment. A false positive test was defined as an elevated IgG4/IgG RNA ratio ≥5% in combination with a histopathologically proven malignancy or clear evidence of a benign disease different from IgG4-RD. A false negative test was defined as an IgG4/IgG RNA ratio <5% and benign histo-/cytopathology with histological features of IgG4-RD (tissue infiltration of >10 IgG4+ plasma cells per high power field (HPF) in biopsy or >50 IgG4+ plasma cells per HPF in organ resection material, in combination with lymphoplasmacytic infiltration, storiform fibrosis and/or obliterative phlebitis^16^) after biopsy/resection for a presumed malignancy. The test was considered true negative if histo-/cytopathology showed signs of malignancy or clear evidence of a benign disease different from IgG4-RD in combination with an IgG4/IgG RNA ratio ≤5%. Statistical differences between benign and malignant disease for serum levels of IgG4, CA 19.9 and blood IgG4/IgG RNA ratio were evaluated using Wilcoxon-Mann-Whitney test. All statistical testing was 2-sided and performed at the 0.05 significance level (*p* <0.05) using SPSS version 25 (IBM SPSS statistics).

The study protocol was approved by the Medical Research and Ethics Committee of the Amsterdam UMC, location AMC (W18_263 # 18.328).

## Results

A total of 213 consecutive patients presenting at a specialized multidisciplinary hepato-pancreato-biliary outpatient clinic for suspicion of pancreatobiliary malignancy were investigated between February and September, 2019. One hundred and four patients (48.8%) were male and 109 (51.2%) were female, with a mean (±SD) age of 68 ± 11 years. Demographic, clinical and biochemical characteristics are summarized in Table 1. A benign disease was diagnosed in 35 patients (16.4%): 7 patients were diagnosed with cholecystitis and/or cholecysto-/choledocholithiasis, 7 patients with chronic pancreatitis or AIP type 2, 10 patients with a pancreas cyst, and 8 patients with other benign diseases or no abnormalities. In this cohort no patients were diagnosed with PSC. Based on clinical, laboratory and imaging findings, 3 patients (1.4%) were diagnosed with IgG4-RD. All 3 patients were male with a mean age of 74 years, the blood IgG4/IgG RNA ratio was elevated (resp. 10.9%, 37.9% and 41.2%) and in 2 of the 3 patients serum IgG4 was more than 10× elevated (>14.0 g/L). Two patients were treated with prednisolone and subsequently with azathioprine and responded to prednisolone by rapid improvement of laboratory tests and, at imaging, decrease of tumor size and/or bile duct dilatation. One patient suffering from dysregulated diabetes was treated with ursodeoxycholic acid with marked improvement of cholestasis as well. A total of 178 patients were diagnosed with a malignancy, of whom 165 patients had a primary malignancy of the pancreas or biliary tract: 110 patients (51.6%) were diagnosed with pancreatic cancer, 37 patients (17.4%) with cholangiocarcinoma, 11 patients (5.2%) with carcinoma of the papilla of Vater and 7 patients (3.3%) with gallbladder carcinoma.

**Table 1:** Clinical and biochemical characteristics of 213 consecutive patients presenting with suspected or proven hepato-pancreato-biliary malignancies.

Characteristics	
Age, years [mean ± SD]	68 ± 11
Sex, male/female	104 (49%)/109 (51%)
Total bilirubin, μmol/L	15 (8–95)
Alkaline phosphatase, U/L	175 (85–395)
Gamma-glutamyltransferase, U/L	124 (35–511)
Aspartate aminotransferase, U/L	37 (22–85)
Alanine aminotransferase, U/L	51 (22–117)
IgG4/IgG RNA, %	3.9 (2.0–8.0)
IgG4, g/L	0.48 (0.25–0.88)
Carbohydrate antigen 19.9, kU/L	127 (19–1,235)

Results of laboratory tests are presented as medians (IQR).

**Fig. 1:**
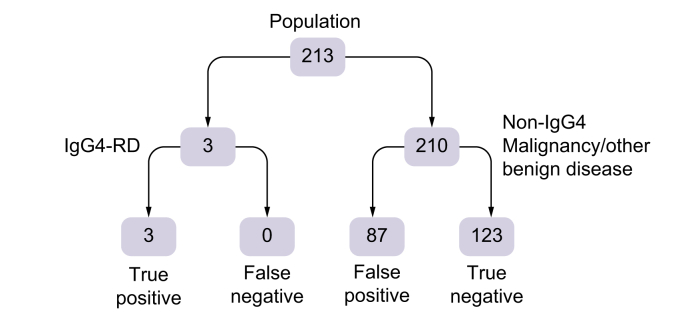
Limited value of elevated blood IgG4/IgG RNA ratio (threshold 5%) in the prospective DIPAC trial to identify patients with IgG4-RD among a patient cohort suspected or proven to suffer from hepato-pancreato-biliary malignancy. Sensitivity was 100%, specificity was 58.6%, the positive predictive value was 3.3%. CA 19.9, carbohydrate antigen 19.9; IgG4-RD, IgG4-related disease.

The blood IgG4/IgG RNA ratio was true positive in 3 patients with IgG4-RD (3.3%) and false positive (median IgG4/IgG RNA ratio 9.8%, IQR 6.3–15.2) in 87 patients (40.8%) with other benign or malignant disease. In 123 patients (58.6%) the test was true negative (Fig. 1). Receiver operating characteristics (ROC) curve analysis revealed an area under the curve (AUC) for IgG4/IgG RNA ratio of 0.793 to predict IgG4-related disease (sensitivity 100%, specificity 58.6%). No differences were observed in patient characteristics (age, gender, symptoms at presentation, biochemical tests, diagnosis) between patients with a false positive test (n = 87) in comparison to patients with a true negative test (n = 123) (Table 2).

**Table 2:** Comparison of characteristics of patients tested true negative and false positive using IgG4/IgG RNA ratio in blood.

Patient characteristics	True negative n = 123	False positive n = 87	*p* value∗
Gender, n (%)			0.51
Female	61 (49.6%)	48 (55.2%)	
Male	62 (50.4%)	39 (44.8%)	
Diagnosis, n (%)			0.08
Pancreatic carcinoma	61 (49.6%)	49 (56.3%)	
Cholangiocarcinoma	26 (21.1%)	11 (12.6%)	
Galbladder carcinoma	6 (4.9%)	1 (1.1%)	
Papil carcinoma	4 (3.3%)	7 (8.0%)	
Primary malignancy unknown	0 (0%)	2 (2.3%)	
Other malignancy	5 (4.1%)	6 (6.9%)	
Benign disease	21 (17.1%)	11 (12.6%)	
Symptoms, n (%)			
Jaundice	47 (38.2%)	28 (32.2%)	0.45
Abdominal discomfort	80 (65.0%)	48 (55.2%)	0.19
Pruritus	23 (18.7%)	26 (29.9%)	0.09
Fever	3 (2.4%)	0 (0%)	0.38
Decreased appetite	39 (31.7%)	24 (27.6%)	0.63
Weight loss	67 (54.5%)	51 (58.6%)	0.48
Age, mean (SD)	67.4 (11.8)	67.7 (11.0)	0.85
Biochemical tests, mean (SD)			
C-reactive protein	19.9 (29.0)	17.7 (29.2)	0.59
Bilirubin	88.3 (142)	95.1 (146)	0.74
Alkaline phosphatase	306 (318)	297 (321)	0.85
Gamma-glutamyltransferase	364 (511)	353 (541)	0.88
Aspartate aminotransferase	68 (74)	71 (77)	0.78
Alanine aminotransferase	103 (150)	112 (160)	0.67
Carbohydrate antigen 19.9	2,110 (8,160)	2,200 (7,170)	0.94
IgG4	0.59 (0.55)	0.69 (0.58)	0.25

Categorical variables are presented as n (%). Continuous variables are presented as mean (SD).

∗ Continuous variables have been tested using Student's *t* test, categorical variables have been tested using Chi-square test.

CA19.9 differentiated benign and malignant diseases (*p* <0.01), but no difference in serum IgG4 or blood IgG4/IgG RNA ratio (*p* = 0.6, each) between these groups was observed (Table 3). An AUC for CA19.9 of 0.721 to predict malignant disease in this cohort (sensitivity 73.6%, specificity 70.6%) was reached.

**Table 3:** Discriminant function of laboratory tests for benign *vs.* malignant hepato-pancreato-biliary disease in 213 consecutive patients presented at a specialized multidisciplinary outpatient clinic.

	Benign disease (n = 35)	Malignant disease (n = 178)	*p* value
IgG4/IgG RNA ratio, %	4.0 (2.1–6.9)	3.9 (2.0–8.2)	0.6
IgG4, g/L	0.4 (0.3–1.1)	0.5 (0.2–0.9)	0.6
CA 19.9, kU/L	16.5 (7–50)	201 (33–1,646)	<0.01

Results are presented as medians (IQR); Wilcoxon-Mann-Whitney test.

## Discussion

In patients presenting at a dedicated outpatient clinic with suspected or proven biliary or pancreatic cancer, the accuracy of an elevated IgG4/IgG RNA ratio to discriminate pancreatic and biliary cancer from IgG4-RD was poor, as demonstrated in this open prospective DIPAC trial. The results of the interim analysis led us to stop the trial and advise against the use of this test for discrimination of IgG4-RD from biliary and pancreatic malignancies outside a research setting. Retrospective re-analysis of a large cohort of hepato-pancreato-biliary patients is underway to understand the discrepancy between previous^12^ and actual results regarding a diagnostic value of the blood IgG4/IgG RNA ratio.

## Financial support

Funding provided by the Dutch Digestive Foundation (10.13039/501100008359MLDS) to UB.

## Authors' contributions

Based on CRediT. EV – Data curation, Formal analysis, Investigation, Project administration, Writing (original draft); FT – Data curation, Formal analysis, Investigation, Project administration, Writing (original draft); LH – Conceptualization, Funding acquisition, Writing (review & editing); JH – Data curation, Project administration, Software; NM – Software, Validation, Visualization, Writing (review & editing); JV – Investigation, Writing (review & editing); JvHo – Project administration, Resources, Writing (review & editing); MB – Writing (review & editing); PF – Resources, Writing (review & editing); NV – Conceptualization, Methodology, Supervision, Writing (review & editing); UB – Conceptualization, Data curation, Formal analysis, Funding acquisition, Investigation, Methodology, Project administration, Resources, Supervision, Writing (original draft), Writing (review & editing).

## Conflict of interest

The authors declare no conflicts of interest that pertain to this work.

Please refer to the accompanying ICMJE disclosure forms for further details.

## Supplementary data

CTAT table.pdf
