# Supplementary material for: IgG4/IgG RNA ratio does not accurately discriminate IgG4-related disease from pancreatobiliary cancer
Source: JHEP Rep. 2020 Apr 14;2(4):100116. doi: 10.1016/j.jhepr.2020.100116 (PMC7332528; doi:10.1016/j.jhepr.2020.100116)
Supplement: CTAT table.pdf [file mmc1.pdf]

JHEP|Reports

JHEP Reports

CTAT methods

Tables for a "Complete, Transparent, Accurate and Timely account" (CTAT) are now mandatory for all revised submissions. The aim is to enhance the reproducibility of methods.

- Only include the parts relevant to your study
- Refer to the CTAT in the main text as 'Supplementary CTAT Table'
- Do not add subheadings
- Add as many rows as needed to include all information
- Only include one item per row

If the CTAT form is not relevant to your study, please outline the reasons why:

1.1 Antibodies

Name	Citation	Supplier	Cat no.	Clone no.
N.A.				

1.2 Cell lines

Name	Citation	Supplier	Cat no.	Passage no.	Authentication test method
N.A.					

1.3 Organisms

Name	Citation	Supplier	Strain	Sex	Age	Overall n number
N.A.						

1.4 Sequence based reagents

Name	Sequence	Supplier
one universal Ig forward primer,<br><br>one generic IgG reverse primer,<br><br>one specific IgG4 reverse primer	(5′-GCTGCCTGGTCAAG GACTAC-3′)<br><br>(5′-TCTTGTCCACCTTGGT GTTG-3′)<br><br>(5′-TACGTTGCAGGTGTA GGTCTTC-3′)	

1.5 Biological samples

Description	Source	Identifier
N.A.		

1.6 Deposited data

Name of repository	Identifier	Link
N.A.		

1.7 Software

Software name	Manufacturer	Version
LinRegPCR software15		

1.8 Other (*e.g.* drugs, proteins, vectors etc.)

SensiFAST SYBR Lo-ROX reagent	GC Biotech, Alphen aan den Rijn, Netherlands	catalog no. BIO-94005, Bioline;

1.9 Please provide the details of the corresponding methods author for the manuscript:

Prof. Dr. Niek de Vries, Department of Rheumatology & Clinical Immunology,

Amsterdam UMC, University of Amsterdam;

email: n.devries1@amsterdamumc.nl

2.0 Please confirm for randomised controlled trials all versions of the clinical protocol are included in the submission. These will be published online as supplementary information.

N.A.
